# Cost and Life Cycle Emissions of Ethanol Produced with an Oxyfuel Boiler and Carbon Capture and Storage

**DOI:** 10.1021/acs.est.2c04784

**Published:** 2023-03-21

**Authors:** John Dees, Kafayat Oke, Hannah Goldstein, Sean T. McCoy, Daniel L. Sanchez, A. J. Simon, Wenqin Li

**Affiliations:** † Energy and Resources Group, University of California, Berkeley, 345 Giannini Hall, Berkeley, California 94720, United States; ‡ Department of Chemical and Petroleum Engineering, University of Calgary, 750 Campus Dr NW, Calgary, AB T2N 4H9, Canada; § Lawrence Livermore National Laboratory, 7000 East Avenue, Livermore, California 94550, United States; ∥ Environmental Science, Policy, and Management (ESPM), University of California, Berkeley, 130 Mulford Hall #3114, Berkeley, California 94720, United States

**Keywords:** ethanol, life cycle assessment, CCS, techno-economic analysis, oxycombustion, carbon negative fuel

## Abstract

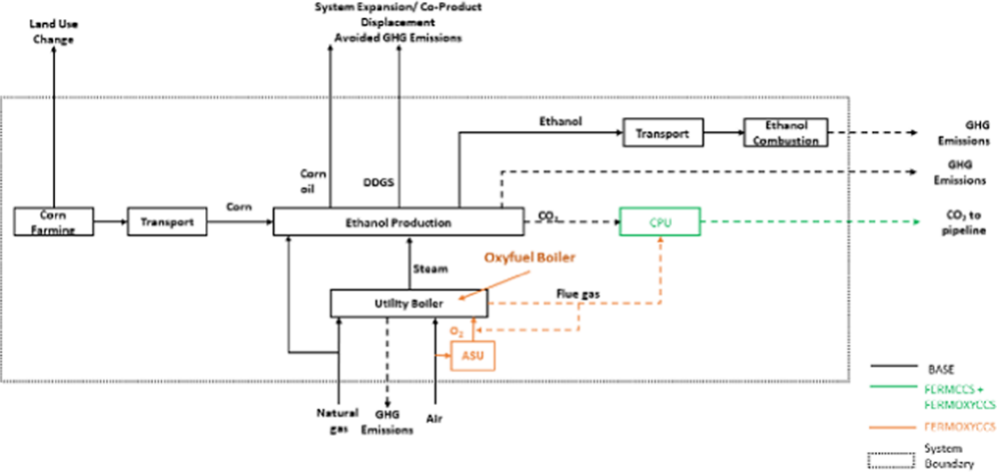

Decarbonization of transportation fuels represents one of the most vexing challenges for climate change mitigation. Biofuels derived from corn starch have offered modest life cycle greenhouse gas (GHG) emissions reductions over fossil fuels. Here we show that capture and storage of CO_2_ emissions from corn ethanol fermentation achieves ∼58% reduction in the GHG intensity (CI) of ethanol at a levelized cost of 52 $/tCO_2_e abated. The integration of an oxyfuel boiler enables further CO_2_ capture at modest cost. This system yields a 75% reduction in CI to 15 gCO_2_e/MJ at a minimum ethanol selling price (MESP) of $2.24/gallon ($0.59/L), a $0.31/gallon ($0.08/L) increase relative to the baseline no intervention case. The levelized cost of carbon abatement is 84 $/tCO_2_e. Sensitivity analysis reveals that carbon-neutral or even carbon-negative ethanol can be achieved when oxyfuel carbon capture is stacked with low-CI alternatives to grid power and fossil natural gas. Conservatively, fermentation and oxyfuel CCS can reduce the CI of conventional ethanol by a net 44–50 gCO_2_/MJ. Full implementation of interventions explored in the sensitivity analysis would reduce CI by net 79–85 gCO_2_/MJ. Integrated oxyfuel and fermentation CCS is shown to be cost-effective under existing U.S. policy, offering near-term abatement opportunities.

## Abstract 2

An oxyfuel boiler can greatly enhance carbon capture and storage potential at conventional ethanol refineries at costs amenable to existing U.S. policy support.

## 1 Introduction

Carbon dioxide emissions from the power, transport, and industrial sectors are key drivers of anthropogenic climate change.^1^ Efforts to limit global anthropogenic warming to 2 °C by 2100 have spurred efforts to decarbonize these sectors and eliminate emissions from fossil fuels. One solution in the mitigation portfolio is the use of biomass as an alternative fuel or feedstock that displaces use of fossil fuels and fossil-based products and, if biomass is sustainably produced, results in an overall emissions reduction. Sustainable biomass supplies are limited; thus, energy transition models tend to rely on electrification and efficiency where possible with a targeted role for biomass, primarily in the transportation sector.^2−4^ Biofuels can be a low-carbon alternative in challenging sectors such as heavy transport, steel, cement, and aviation and can assist in decarbonizing light-duty transportation alongside vehicle electrification in the near-term.^5^ When combined with capture and storage (CCS) of high-purity CO_2_ streams made available during the conversion of biomass to liquid fuels, the carbon intensity of biofuels can be driven lower or in some cases achieve net removal of carbon from the atmosphere.^6^

Biobased ethanol represents a significant component of the transportation fuel mix in the United States and Brazil (4%^7^ and 20%^8^ by energy content, respectively). Recent research has highlighted near-term opportunities to develop CCS capabilities for existing ethanol capacity.^9,10^ In the U.S., approximately 15.8 billion gallons (59.8 billion liters) of ethanol, primarily from corn, are produced annually for blending with gasoline.^11^ An estimated 45 Mt/yr of high-purity CO_2_ generated from fermentation is available for capture at these facilities.^9^ Fermentation CO_2_ is considered “low-hanging fruit” due to the relative purity of the CO_2_ stream. Similarly, Brazil consumes 7.4 billion gallons (28 billion liters) of fuel ethanol, primarily derived from sugarcane^12^ but with a growing contribution from corn.^13^ The fermentation CO_2_ capture potential at Brazilian ethanol facilities is as high as 28 Mt CO_2_/y.^14^ There is also considerable interest in upgrading ethanol and other alcohol-based fuels into sustainable aviation fuels, at high energy and carbon conversion efficiency.^15^

Carbon dioxide from fermentation can be captured at a relatively low cost, requiring only dehydration and compression.^16^ Unlike other CO_2_ point sources, ethanol production generates a high purity (99%) stream of fermentation CO_2_ containing only CO_2_, H_2_O, and small amounts of sulfur and organic compounds.^17,18^ The technical feasibility of fermentation CCS and permanent geologic storage in saline aquifers has been demonstrated at one U.S. site owned by ADM where captured CO_2_ was sequestered in the Mt. Simon Sandstone formation:^19^ additional projects are proposed, some interconnected by common-carrier CO_2_ pipelines.^20−24^ There is a growing literature around CCS in the Brazilian ethanol context, as well.^10,14^

Policy support is key to the development of low-carbon biobased fuels. In the United States, production volumes are largely supported by the Renewable Standard (RFS), which established annual biofuel blending requirements that result in approximately 10% blend of ethanol in most gasoline used in light-duty transport.^25^ Continued improvement in the CI of ethanol has largely been driven by performance-based policies implemented at the state level such as California’s Low Carbon Fuel Standard (LCFS)^26^ and both federal and state policies supporting the deployment of CCS.^26,27^ Brazil’s ethanol industry has been supported by blending requirements as well. These requirements have varied since the implementation of the Brazilian National Alcohol Program (Proálcool) in 1975. In addition to tax incentives driving large-scale adoption of flex fuel vehicles since the early 2000s, more recently, Law No. 12,490 (2011) set ethanol blending requirements at 18%, and the executive branch has adjusted volumes as high as 27% in recent years.^28,29^ Brazil’s adoption of the RenovaBio policy (2017) is of particular import as there is now a performance-based market mechanism at the national level for low-CI biofuels analogous to the LCFS program.^30^ In these policy contexts, interventions such as CCS can substantially reduce the carbon intensity of ethanol while providing the necessary revenue support to compete with conventional fuels, learn-by-doing, and ultimately bring down costs. There is potential to not only reduce the climate impact of current light-duty transport but can also provide low-carbon feedstocks to chemicals manufacturing or sustainable aviation fuel, a rapidly growing market, with some market research firms estimating a compound annual growth rate (CAGR) of 60% or more through 2030.^31^

The above context motivates exploration of interventions to reduce the CI of ethanol beyond capture and storage of CO_2_ from fermentation. Researchers and operators have already explored many options. Switching from first-generation starch and sugar feedstocks to second-generation cellulosic feedstocks has clear CI benefits, as these feedstocks typically have much lower production emissions and less concern regarding emissions from land use change. However, there remain substantial technological barriers to make cellulosic ethanol cost-effective.^32−35^ Other interventions target process engineering and facility operations to achieve higher efficiencies and protect equipment functionality. Improved boiler and condenser integration, high gravity fermentation, pervaporation membranes, substitution of dewatering processes, multieffect distillation, and mechanical vapor recompression in the distillation column are examples of potential interventions.^36−38^

The heat and power requirements of a corn ethanol facility typically represent a substantial fraction of emissions and a concurrent opportunity to decarbonize the industry. Sugarcane and cellulosic ethanol facilities substantially improve ethanol CI by utilizing cellulosic wastes/residues as a biogenic source of fuel for heat and power needs.^32,39,40^ However, conventional corn and sugar beet ethanol facilities often rely on fossil-fuel boilers and grid power to supply process heat and electricity. Only one study, to our knowledge, has explored the potential for capture and storage of carbon from fossil co-generation at conventional ethanol refineries from conventional boilers.^41^ This earlier study considered use of a first-generation (monoethanolamine or MEA) solvent for post-combustion capture from onsite heat and electricity power generation for the production of ethanol from sugar beets. This reflects a significantly different route to ethanol production than is dominant in North America. Moreover, in this case, the capture process absorbs CO_2_ in aqueous solution, requiring substantial heat inputs for the regeneration of the capture solvent. The combustion of additional natural gas to meet this demand results in an increase in nonrenewable energy consumption and a penalty on emissions reductions.^41^ As such, alternatives to solvent capture of diffuse post-combustion CO_2_ streams have been proposed.^42−44^

Oxyfuel combustion is one potential alternative to solvent-based post-combustion capture. In an oxyfuel process, high-purity oxygen takes the place of ambient air in the combustion vessel, greatly reducing the volume of nitrogen and other species in combustion resulting in a high-purity CO_2_ stream in the combustion products. Oxyfuel process designs have been studied and demonstrated in the fossil fuel power,^45−48^ petrochemical,^49^ cement,^50^ and steel^51^ industries. While it is not considered commercial (e.g., TRL 9) at the scale of a large power plant,^52^ demonstrations of the technology have been undertaken at the scale of the boiler used in an ethanol mill (e.g., 30–50 MW_th_). In this context, one benefit of oxyfuel combustion is that the energy requirements for capture are largely electrical, which means that the system can benefit from decreasing electricity grid CI over time (or be directly served by renewable generation). Moreover, an oxyfuel boiler does not have conventional “stack” emissions. However, the resulting reduction in air emissions may come at the cost of increased amounts of solid or liquid waste.^53^ Operational data on criteria pollutants from natural gas oxyfuel boilers is limited but boilers can likely meet regulatory limits in the United States.^54^

This analysis explores oxyfuel combustion combined with CCS to address boiler emissions in a corn-based ethanol plant. We propose the integration of an oxyfuel natural gas boiler to supply refinery heat demand. In this process design, natural gas is combusted in high-purity oxygen (95–99%) with a fraction of the flue gas recycled to the boiler to control combustion temperature. An air separation unit (ASU) is required to supply oxygen for oxycombustion. The flue gas is composed primarily of water and CO_2_ making the flue gas stream compatible with the fermentation CO_2_ stream, allowing greater process integration and dehydration in the same CO_2_ purification unit (CPU). To our knowledge, this is the first analysis of potential integration of oxyfuel combustion in the production of ethanol combined with CCS.

Here we estimate the emissions mitigation benefits and costs of integrating fermentation and oxyfuel boiler CCS to produce low-carbon corn ethanol. We consider a conventional dry mill corn ethanol facility located in the Midwestern United States. We calculate the well-to-wheel life cycle carbon intensity (CI) and production costs of two intervention scenarios: (1) fermentation CO_2_ capture only and (2) fermentation and oxyfuel CO_2_ capture. Cost estimates are presented without policy incentives to estimate minimum ethanol selling price (MESP) and unit cost of carbon abatement. Key life cycle input and cost sensitivities as well as MESP sensitivity to existing policy support such as California’s LCFS program and the U.S. 45Q tax credit are presented in the final section. Our analysis tests the hypothesis that oxyfuel combustion is a cost-effective option to decarbonize corn ethanol production under existing policy regimes.

## 2 Materials and Methods

### 2.1 Baseline Facility

The baseline facility (BASE) for this study is assumed to be a modern dry mill ethanol refinery in the midwestern United States with a capacity of 40 M-gal (151 ML) of ethanol per year. The Midwest is home to a high density of existing corn production and ethanol refineries, and parts of the region are proximate to suitable formations for geologic sequestration of CO_2_ such as the Forest City and Illinois Basins.^19,55^ The facility produces dried distiller’s grains and solids (DDGS) and corn oil co-products. BASE utilizes a conventional natural gas boiler for thermal energy requirements and utilizes a direct natural gas-fired drying system for the DDGS co-product. This drying configuration is a conservative choice, as the selection of an indirect steam dry system will make more CO_2_ available for capture from the boiler. We explore the steam dry option in the Sensitivity Analysis section and the Supporting Information (SI). Electricity is supplied by the Midwestern Reliability Organization (MRO) for which we assume 2019 grid average emissions and costs. BASE life cycle inventory data is consistent with Argonne National Lab’s GREET.net 2019 model,^56^ except for power and heat demand and the relative ethanol and co-product yields, which are adjusted to match our own Aspen Plus model results. BASE energy demand is based on Mueller’s 2008 report which reports an average natural gas thermal energy requirement for dry grind refineries of 29,009 btu/gal (8.1 MJ/L) (HHV) and 0.73 kWh/gal (0.19 kWh/L) electricity requirement.^57^ Approximately 62% of the thermal energy requirement is steam, equivalent to a thermal duty of 24,427 kW_th_. Corn is assumed to travel an average of 50 miles (80.5 km) by heavy diesel truck to the ethanol refinery. Ethanol travels an additional 50 miles by heavy truck for denaturing and blending into transport fuel. The facility is assumed to operate 7882 h per year.

### 2.2 Fermentation CO_2_ Capture

For the fermentation-only CCS (FERMCCS) scenario, we performed a full material balance to determine the quantity of CO_2_ capturable from a 40 M-gal (151 ML) per year ethanol plant. The composition of corn is reviewed from several literature sources^58−60^ and given in the Supporting Information (see Table S1). Fermentation is assumed to have 93.2% conversion efficiency, while liquefaction and saccharification conversion efficiency and ethanol recovery is 99%. Corn is assumed to be composed of 40.5% carbon. The density of ethanol is 0.79 kg/L. The reaction equations are given in Supporting Information S1.1. Overall yield from 1 kg corn is 0.33 kg ethanol, 0.28 kg DDGS, 0.01 kg corn oil, and 0.32 kg CO_2_. Fermentation CO_2_ is captured at a rate of 13,089 kg/h and assumed to be at 100% purity. Fermentation CO_2_ is dehydrated, compressed, liquefied, and pumped at 150 bar, which is assumed to be sufficient to transport the gas by pipeline 100 miles to geologic storage without need for further compression. This is carried out by the CO_2_ processing unit (CPU) and modeled using Aspen Plus V11. The additional electricity demand for the CPU is estimated to be 110 kWh/t CO_2_ using this model.

### 2.3 Integration of the Oxyfuel Boiler with CO_2_ Capture

For the integrated oxyfuel CCS scenario (FERMOXYCCS), we modeled the steam requirement of the BASE plant to be supplied by the oxyfuel boiler, with integrated capture of the CO_2_ streams produced during the combustion and fermentation steps. We modeled additional power requirements for oxygen provision by the ASU and for handling additional CO_2_ throughput in the CPU. The overall additional power requirement is 2730 Btu/gal (0.76 MJ/L) of ethanol. An additional 5056 kg CO_2_/h is captured from the oxyfuel boiler, assuming a 98% capture rate. Energy and carbon balance results from the Aspen model can be found in Supporting Information S1.2.

**Figure 1:**
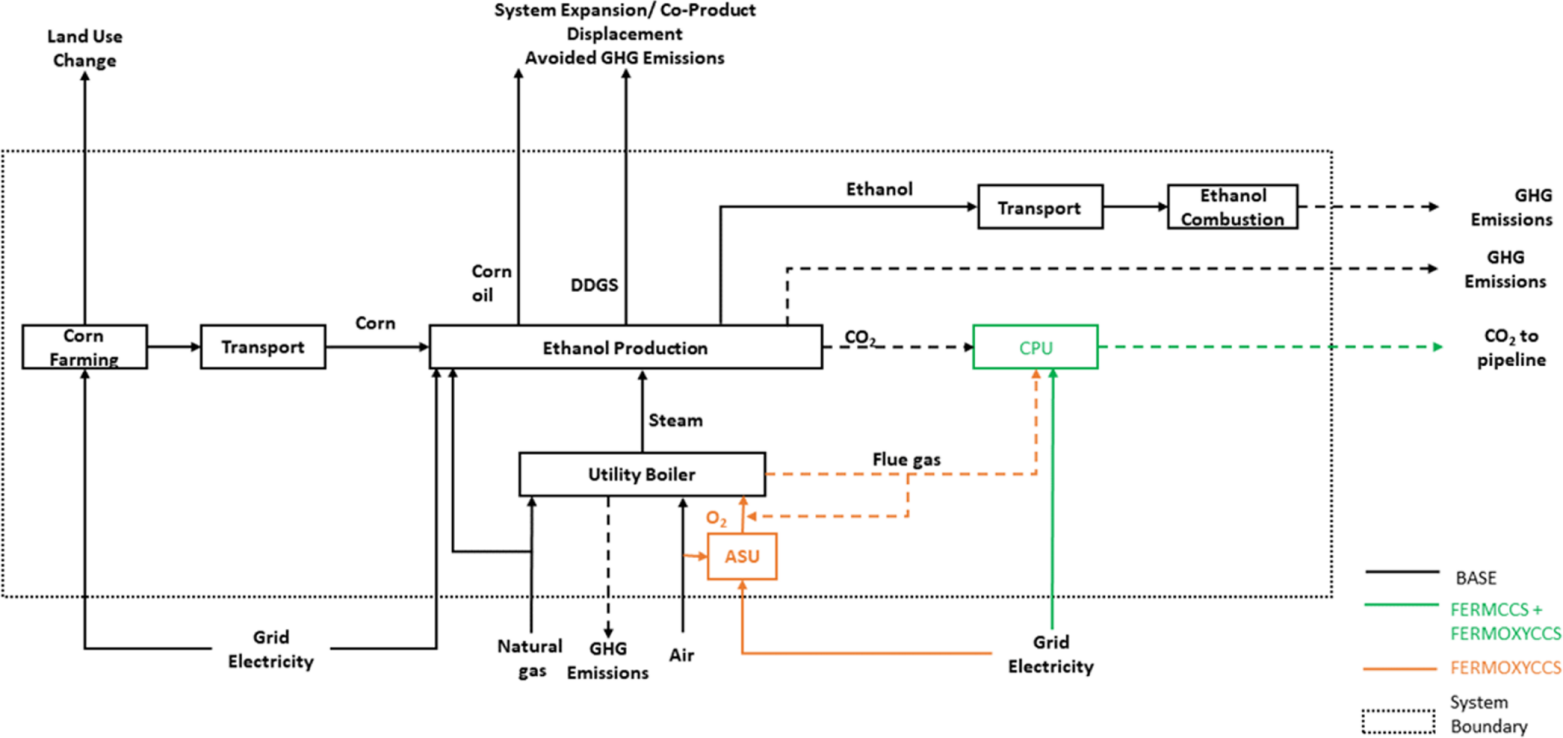
Process configuration for integration of fermentation CCS (FERMCCS) and the oxyfuel boiler (FERMOXYCCS) with the BASE facility. The dashed box represents the system boundary for the LCA. Land use change and co-product displacement are handled via system expansion. DDGS = dry distillers grains and solids, CPU = CO_2_ processing unit, ASU = air separation unit.

Figure 1 shows a block-flow representation of the FERMCCS and FERMOXYCCS processes with the BASE plant. In the FERMOXYCCS case, steam requirements are supplied by an oxyfuel utility boiler. Oxygen is separated from air by cryogenic distillation in the ASU and is used for combustion of fuel in the oxycombustion unit for steam generation. The combustion stream joins the fermentation stream. In both CCS cases, the CO_2_ is sent to the CPU for final clean-up and compression prior to pipeline transportation.

### 2.4 Techno-Economic Assessment

We perform a techno-economic assessment (TEA) to determine the minimum ethanol selling price (MESP) for each of the scenarios and cost sensitivity cases. The TEA is informed by a (1) conceptual-level process design based on research data, rigorous material and energy balance calculations via commercial simulation tools such as Aspen Plus, (2) capital and project cost estimations using an in-house model, (3) and a discounted cash flow economic model used to determine MESP.

We adapted an in-house version of the United States Department of Agriculture (USDA) Dry Mill Ethanol Production to serve as the basis for our TEA. This model is utilized and regularly updated by the National Renewable Energy Laboratory (NREL).^61,62^ This is a capacity factored model that uses flow rates and equipment duties to estimate the purchased cost of equipment based on reference costs and applies an installation factor to arrive the installed or inside battery limit (ISBL) capital cost. The reference costs are primarily based on detailed equipment costs reported in previous NREL cost assessments.^61−65^ The operating expense (OPEX) calculations are also based on material and energy balance calculations using process simulations and are consistent with previously developed TEA models.^62−65^ Raw materials include feedstocks, chemicals, catalysts, and utilities. All costs are adjusted to 2020 U.S. dollars using the U.S. Bureau of Labor Statistics’s Labor Cost Index^66^ and Chemical Cost Index^67^ as well as the Chemical Engineering Plant Cost Index.^68^

We perform a discounted cash flow analysis using the financial assumptions shown in Table 1. The MESP is the minimum fuel selling price necessary to generate a net present value of zero assuming a 10% after-tax return on equity.

**Table 1:** Main Assumptions of Economic Analysis

economic parameters	assumed basis
basis year for analysis	2020
debt/equity for plant financing	60%/40%
interest rate and term for debt financing	8%/10 years
internal rate of return for equity financing	10%
total income tax rate	21%
plant life	20 years
construction period	3 years
fixed capital expenditure schedule (years 1–3)	32% in year 1, 60% in year 2, 8% in year 3
start-up time	0.5 year
revenues during start-up	50%
variable costs during start-up	75%
fixed costs during start-up	100%
outside battery limit (OSBL) costs	10.5% of ISBL
total installed cost (TIC)	total of ISBL and OSBL costs
indirect costs	% TIC
prorated expenses	10%
home office and construction fees	25%
field expenses	10%
project contingency	10%
total plant cost (TPC)	TIC + indirect costs
other costs (start-up and permitting)	10% TPC
total capital investment (TCI)	TPC + other costs
working capital	5% TPI

Table 2 shows estimated capital costs, operating costs, and product prices used in the cash flow analysis to calculate the MESP. Feedstock, electricity, fuel costs, and co-product selling prices are scaled to 2020 dollars from costs representative of a 2016 base year. The CO_2_ capture costs were scaled from reported costs from the Archer Daniel Midland Demonstration in Decatur, IL^69^ based on the Aspen Plus energy and mass balance. Similarly, the ASU costs and assumptions are scaled from Air Liquide Engineering and Construction Technology Handbook.^70^ No additional plant employee was assumed to run the plant under intervention scenarios. In the FERMOXYCCS scenario, the boiler installation factor was increased from a factor of 3 to 4. Detail on the CO_2_ capture cost model is reported in SI, Section S3.

**Table 2:** Capital and OPEX Assumptions and Costs (2020 USD Basis)

capital costs	
BASE	
total installed equipment cost (ISBL)	$74.5M
total installed cost (TIC)	$82.3M
total plant cost (TPC)	$127.6M
total capital investment (TCI)	$140.3M
CCS and oxyfuel assumptions	
CCS installed cost (ISBL, direct dry cases)	$9M
CCS installed cost (ISBL, direct dry with oxy cases)	$11.2M
ASU installed cost (ISBL, direct dry cases)	$10.6M
CCS utilities and labor (scaled from ADM Decatur, IL)	+33% & +35% of ISBL
OPEX assumptions	
fixed operation costs	$7M/yr
corn	$3.30/bushel
electricity (Midwest)	$0.072/kWh
electricity use for CO_2_ compression (direct dry)	110 kWh/tonne-CO_2_
natural Gas	$4.20/mmBtu ($3.98/GJ)
co-product	Selling price
DDGS	$0.074/lb ($0.163/kg)
corn oil	$0.28/lb ($0.62/kg)

### 2.5 Life Cycle GHG Emissions Analysis

We apply life cycle principles to quantify the incremental change in the well-to-wheel carbon intensity (CI) of corn fuel ethanol from a dry mill ethanol refinery resulting from the integration of CCS and an oxyfuel combustion boiler. We consider the impact of these interventions relative to a BASE refinery where a conventional natural gas-fired industrial boiler is used, and CCS is not employed. The results are not intended to represent a particular ethanol mill but are generally representative of a modern dry mill ethanol facility in the midwestern United States. The life cycle inventory for BASE is drawn from Argonne National Lab’s GREET.net 2019 model (see SI S2.1 for further details).^56^ Ethanol and co-product yield as well as baseline and intervention scenario thermal energy and power requirements have been calculated using Aspen model results and calibrated where necessary to ensure consistency between the techno-economic model and the life cycle inventory.

The functional unit for a life cycle assessment quantifies the function of a product system and is a reference unit for reporting of results (ISO 14040). For this study, life cycle results and comparisons are made on the basis of 1 MJ of ethanol measured as the lower heating value (LHV), as this allows for reasonable comparisons between liquid transportation fuels and conforms to relevant policy contexts such as California’s Low Carbon Fuel Standard.

The system boundary in a life cycle assessment specifies which unit processes are modeled explicitly in the product system (ISO 14044). Clear definition of the boundary is important to assure consistency in product system comparison. For this analysis, the system includes production of corn at the farm, transportation of corn from farm to refinery, production of ethanol from corn starch, and transport of finished ethanol product to blending/denaturing facility (see Figure 1). While we do not consider the impact of blending and denaturing in this analysis, we consider the final combustion of the ethanol and assume that all embodied biogenic carbon returns to the atmosphere at CO_2_.

#### 2.5.1 Treatment of Multifunctionality

Dry mill corn ethanol refineries produce DDGS and often corn oil co-products alongside ethanol. The question arises as to how to allocate emissions and other life cycle impacts between products and co-products. Typical options include system expansion to account for market displacement of co-product alternatives or allocation of life cycle burdens proportionally by energy content, mass, or market value. We opt for system expansion. Ethanol carries all environmental benefits and burdens of production while co-products are assumed to displace similar products in the market. This choice conforms to the practice under the California LCFS program methodology whereby DDGS is assumed to displace alternative agricultural feed. The type and mass of feed displaced relative to the total mass of DDGS are corn (78%), soybean meal (31%), and urea (2.3%). Note that due to displacement ratios greater than 1, the above weight percentages exceed 100%. Corn oil displaces soy oil on a 1:1 basis. Similarly, we adopt system expansion to include direct and indirect land use change (LUC) impacts of corn production, as quantified in the most recent CA-GREET 3.0 model under the LCFS program.

Biogenic CO_2_ emissions are assumed to be “net zero”—that is, we assume that annual crops such as corn will uptake equivalent quantities of CO_2_ in the next growth cycle, thus carbon originating in corn feedstock adds no net CO_2_ to the atmosphere.

## 3 Results and Discussion

We first present the results of the life cycle carbon intensity analysis of BASE, FERMCCS, and FERMOXYCCS scenarios followed by the results of our economic analysis. For benchmarking, we first compare our BASE results to industry data. The approved fuel pathways database for California’s LCFS program reports GHG emissions intensities (CI scores) for corn-only dry mill ethanol facilities ranging between 53 and 86 gCO_2_e/MJ. The mean certified CI is 70.2 gCO_2_e/MJ.^71^ Our BASE scenario yields a CI of 57 gCO_2_e/MJ, comparable to facilities participating in the LCFS program. Corn production is responsible for the largest share of life cycle emissions, followed by onsite natural gas combustion to fire the boiler and dry the DDGS co-product. LUC emissions are the next largest contributor to the CI score followed by electricity generation. Avoided emissions credits awarded for co-product displacement reduce the overall CI in all three scenarios by 11.8 gCO_2_e/MJ. Tailpipe CO_2_ emissions from combustion of the ethanol are assumed to be net zero, due to the biogenic origin of the carbon.

FERMCCS yields a CI of 24 gCO_2_e/MJ, approximately half that of BASE. Emissions from electricity generation increase by 44% due to the extra power required for dehydration and compression of captured CO_2_. Approximately 36 gCO_2_/MJ are captured from the fermentation stage by the CCS system. Onsite combustion of natural gas remains the largest share of onsite facility emissions, accounting for 21 gCO_2_e/MJ.

**Figure 2:**
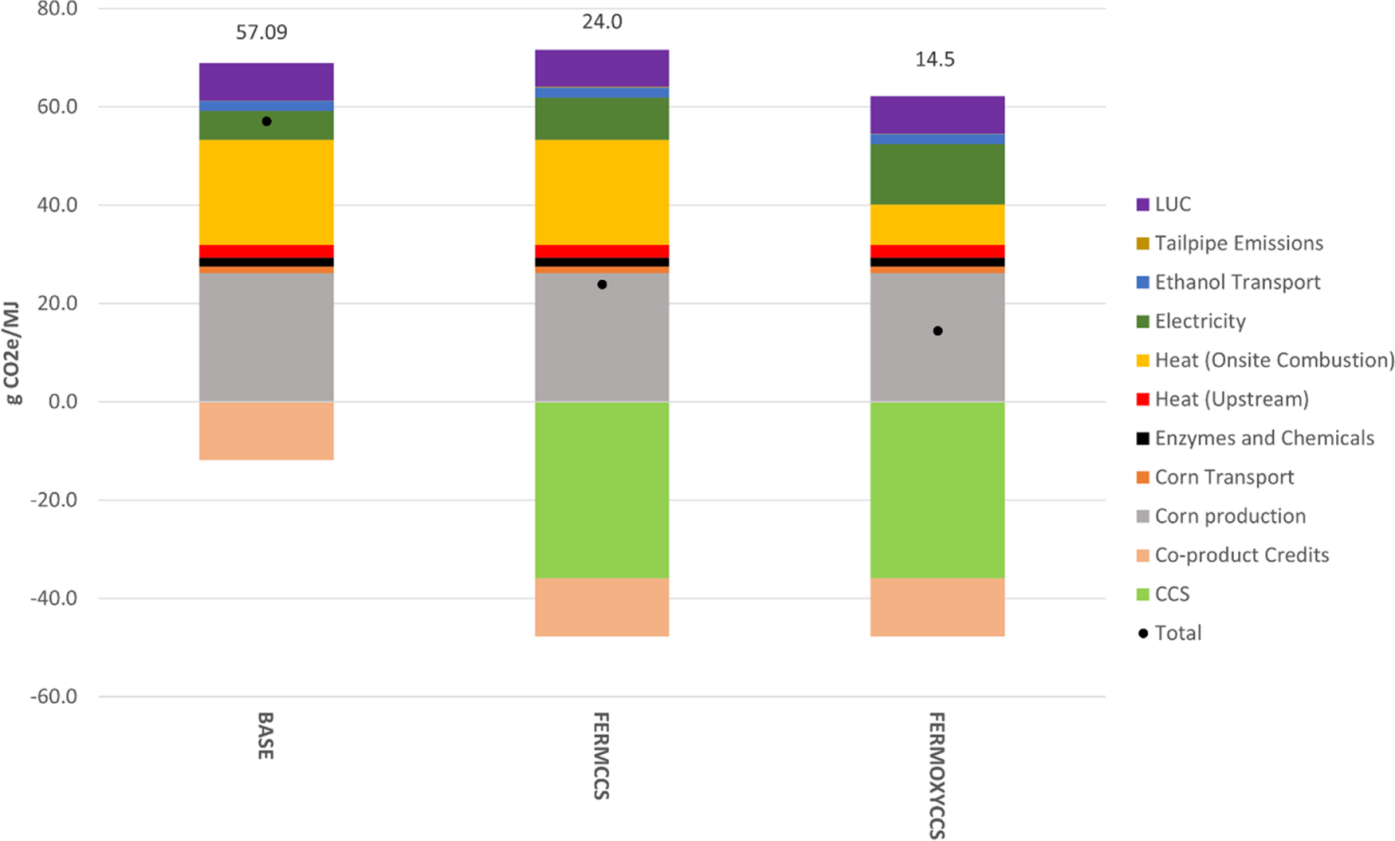
Life cycle carbon intensity (CI) of three ethanol process configurations. BASE = baseline facility with direct drying of DDGS, FERMCCS = CCS on fermentation gas only, FERMOXYCCS = oxyfuel boiler added with CCS on both fermentation and boiler flue gas streams, CCS = carbon capture and sequestration, LUC = land use change (direct + indirect).

FERMOXYCCS targets CO_2_ emissions both from the fermentation column and the oxyfuel boiler. This scenario yields a CI of 15 gCO_2_e/MJ, a 75% reduction from BASE. Additional grid power is required for the ASU and to dehydrate and increased duty on the CPU from the combined fermentation and oxyfuel combustion streams. This results in a 108% increase in emissions from electricity generation. However, the boiler combustion emissions are reduced by 62% through integration of the oxyfuel boiler and the CCS system. The remaining 38% of natural gas combustion emissions are associated with the direct dry DDGS system and are uncaptured in this configuration. An alternative case of indirect steam drying of DDGS allows for capture of most of the emissions from natural gas combustion. We present results for this steam dry scenario in the SI S2.2. However, we preview the CI result in the Sensitivity Analysis section. The captured fermentation CO_2_ remains unchanged in all CCS scenarios at 36 gCO_2_/MJ (Figure 2).

We next assessed the relative costs of CCS in both intervention cases. We benchmarked the MESP for the BASE scenario to the Ethanol Profitability Model developed by Iowa State University Extension Office.^72^ Between January 2020 and December 2021, the model reports monthly average spot prices between $0.77 and $3.12/gallon (multiply by 0.264 to get $USD/L), with an average market price of $1.70/gallon. Production costs over the same period range between $1.81 and $2.03/gallon. The MESP resulting from our TEA of the BASE scenario is $1.93/gallon, comparable to the benchmark estimates.

FERMCCS includes added capital costs from the CPU and additional OPEX costs associated with increased grid power demand and CO_2_ transport and storage. These additional costs result in a MESP of $2.08/gallon. Furthermore, we calculate marginal CO_2_ abatement costs as the ratio between the difference in production cost of the intervention scenario relative to BASE versus the difference in CI relative to BASE. The 58% reduction in CI score in this scenario comes at a cost of $52/tCO_2_e avoided. We compare our estimated costs to IEA estimates for bioethanol CCS, which estimates the breakeven cost between $25 and $35/tCO_2_ captured.^73^ Note, that the cost of CO_2_ captured (and stored) and the cost of CO_2_ abatement are different measures. Our costs reflect the latter metric, which is the cost of the net reduction in emissions resulting from the integration of the CCS system across the life cycle. Additional emissions from grid electricity negate a fraction of the CO_2_ captured; thus, the cost of CO_2_ abated will be greater than the cost of CO_2_ stored. Moreover, the IEA estimate does not include transport and storage cost, which we model at $10/tCO_2_. When these differences are accounted for, our modeled cost is reasonably consistent with the upper range of the IEA estimate.

**Figure 3:**
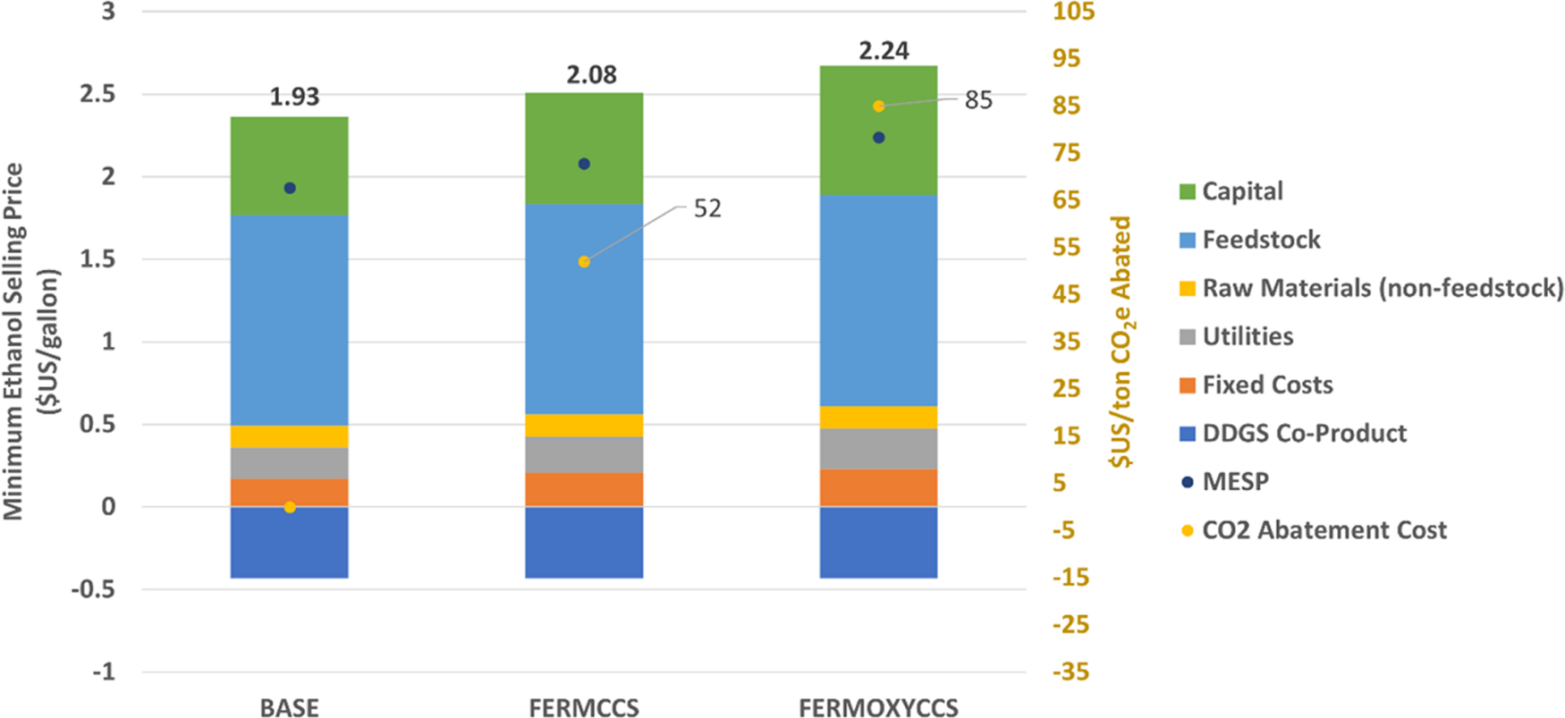
MESP and cost of GHG abatement in the BASE, FERMCCS, and FERMOXYCCS scenarios. There is no abatement or related cost in the BASE case. BASE = baseline facility with direct drying of DDGS, FERMCCS = CCS on fermentation gas only, FERMOXYCCS = oxyfuel boiler added with CCS on both fermentation and boiler flue gas streams, CCS = carbon capture and sequestration. Values shown in U.S. industry standard imperial units. SI values for MESP are $0.51/L (BASE), $0.55/L (FERMCCS), and $0.59/L (FERMOXYCCS). SI values for CO_2_ abatement costs are $57/tonne (FERMCCS) and $94/tonne (FERMOXYCCS).

FERMOXYCCS incurs additional CAPEX for a larger CPU, the ASU, as well as higher costs for the oxyfuel boiler. OPEX increases due to additional power demand as well as additional CO_2_ handling costs. This scenario yields a MESP of $2.24/gallon. The 75% reduction in CI relative to BASE comes at a cost of $85/tCO_2_e avoided. The oxyfuel boiler component of the avoided emissions comes at a cost of $190/tCO_2_e. In this version of the marginal abatement cost calculation, we calculate the change in production costs for FERMOXYCCS relative to FERMCCS only compared to the relative change in CI between FERMCCS and FERMOXYCCS. While this is significantly higher than published estimates of post-combustion capture using conventional methods such as amine solvents estimated to be under $100/tCO_2_,^42,74^ Most capture system cost estimates are for much larger systems (e.g., on the order of 1 MtCO_2_/y) rather than the 139 ktCO_2_/y captured here. In addition, because carbon removal in an oxyfuel boiler comes at the expense of greater electricity use, a lower carbon-intensity grid could improve the cost competitiveness of this approach. We explore this possibility in Section 3.1.2. A comparison of MESP and cost of GHG abatement is shown in Figure 3.

### 3.1 Sensitivity Analysis

#### 3.1.1 Carbon Intensity

**Figure 4:**
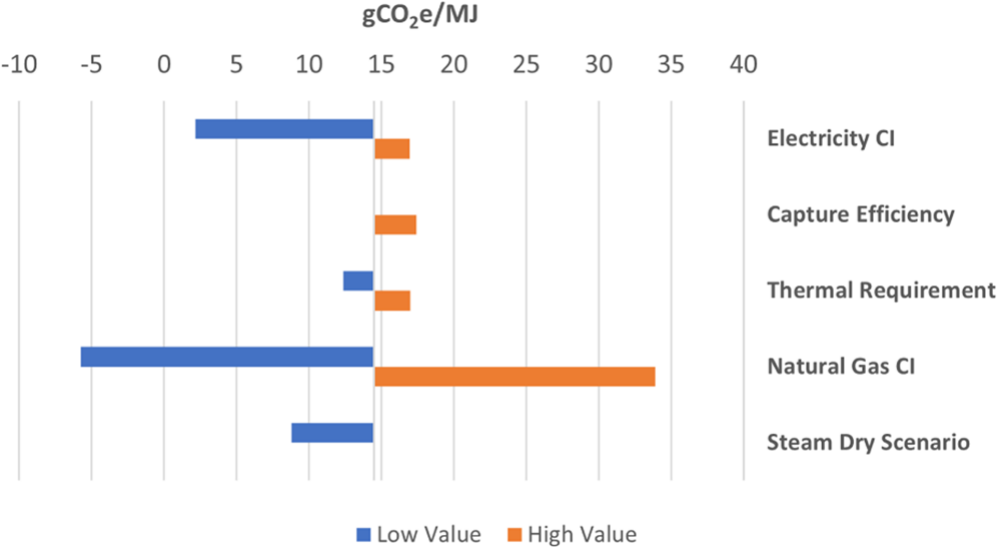
Results of the carbon intensity sensitivity analysis. The Steam Dry case is an alternative configuration that burns all natural gas in the oxyfuel boiler and DDGS is dried indirectly using steam heat. This case is presented alongside the sensitivities for comparison purposes. See SI S2.2 for more details.

Ethanol facilities will differ in geography, process design, and intersection with power and fuel markets. We identified grid carbon intensity, oxyfuel CO_2_ capture efficiency, thermal energy demand, and natural gas CI as key sensitivities to test. We test these sensitivities on FERMOXYCCS only. Results are shown in Figure 4. We omit sensitivities not directly relevant to the oxyfuel and CCS system. The aim is to highlight the incremental benefits and costs of the modeled interventions rather than to precisely model all potential well-to-wheel life cycle scenarios for ethanol.

For electricity, we test a hypothetical zero marginal emissions electricity source and the average distributed U.S. Central/Southern Plains Mix at 730 gCO_2_e/kWh. The latter case is the only average grid CI greater than MROW in GREET and is greater by a factor of 1.2×. In the low-CI test, the CI of ethanol is reduced to 2 gCO_2_e/MJ. The high-end test yields a CI of ethanol of 17 gCO_2_e/MJ.

We also test the capture efficiency of the oxyfuel CO_2_ stream. Capture efficiency performance will be affected by transient operations (e.g., start-up and shut down), during which operations the boiler may be operated on air and the flue gas vented. Boiler capture efficiency is already assumed to be 98%; thus, we do not consider a high-end case. A low-end case where 90% of the CO_2_ from the oxyfuel boiler is captured yields an ethanol CI of 17 gCO_2_e/MJ.

Thermal energy requirements in ethanol facilities have trended downward as reflected in a recent GREET retrospective published by Lee et al.^75^ The low-end thermal energy requirement tested here reflects the 2017 update to GREET model at 26,487 Btu/gal, approximately 9% lower than BASE. The high-end case tests a thermal requirement of 32,043 Btu/gal which is the assumption in the 2016 iteration of the NREL ethanol cost model that served as the basis of the TEA.^62^ This requirement is just over 10% higher than BASE. The thermal energy requirement has a dynamic effect on FERMOXYCCS CI. Upstream natural gas emissions as well as ASU and CPU power demand are positively correlated with increased or decreased thermal requirements. Although BASE boiler emissions are correlated with the thermal requirement, CCS abatement is largely correlated, as well. With respect to the boiler, only the change in leakage (∼2%) as a result of throughput materially impacts the CI sensitivity. The low-end thermal requirement yields a CI of 12 gCO_2_e/MJ. The high-end case yields a CI of 17 gCO_2_e/MJ.

Of the parameters tested, the CI of ethanol is most sensitive to the CI of the boiler fuel. The modeled scenarios assumed natural gas from both North American shale (51.5%) and conventional recovery (48.5%). Methane leakage from the shale portion is assumed to be 0.6% while leakage from the conventional portion is assumed to be just over 2%.^56^ The upstream CI of this natural gas is 7.3 kgCO_2_e/mmBtu. For the low-end estimate, we assume procurement of renewable natural gas (RNG) from landfill gas with an upstream CI of −49.3 kgCO_2_e/mmBtu. The negative value arises from avoided landfill emissions in the GREET model. Recent remote sensing analysis of natural gas recovery in the Permian Basin found methane leakage rates as high as 8%.^76^ For the high-end case, we assume an 8% leakage rate with natural gas procured from conventional recovery only, increasing upstream CI to 61.3 kgCO_2_e/mmBtu. The low-end test case yields an ethanol CI of −6 gCO_2_e/MJ. The high-end case yields and ethanol CI of 34 gCO_2_e/MJ.

In our scenario design, we modeled an alternative process configuration whereby DDGS is dried indirectly by the steam cycle. We present the scenario results here alongside the sensitivity analysis. A full set of results for the steam dry scenario to include a steam dry BASE, FERMCCS, and FERMOXYCCS can be found in SI S2.2. Alternative mass and energy balances can be found throughout the tables in S1.2 under Scenario 2. The essential difference in this scenario is that all natural gas combustion occurs in the oxyfuel boiler for steam generation rather than diverting a portion to a direct dry system. This configuration allows for increased capture of CO_2_ from natural gas combustion. In Figure 4, we show that this configuration is improved relative to the direct dry system with a CI of 9 gCO_2_e/MJ or 39% lower than direct dry FERMOXYCCS and 85% lower than direct dry BASE.

**Figure 5:**
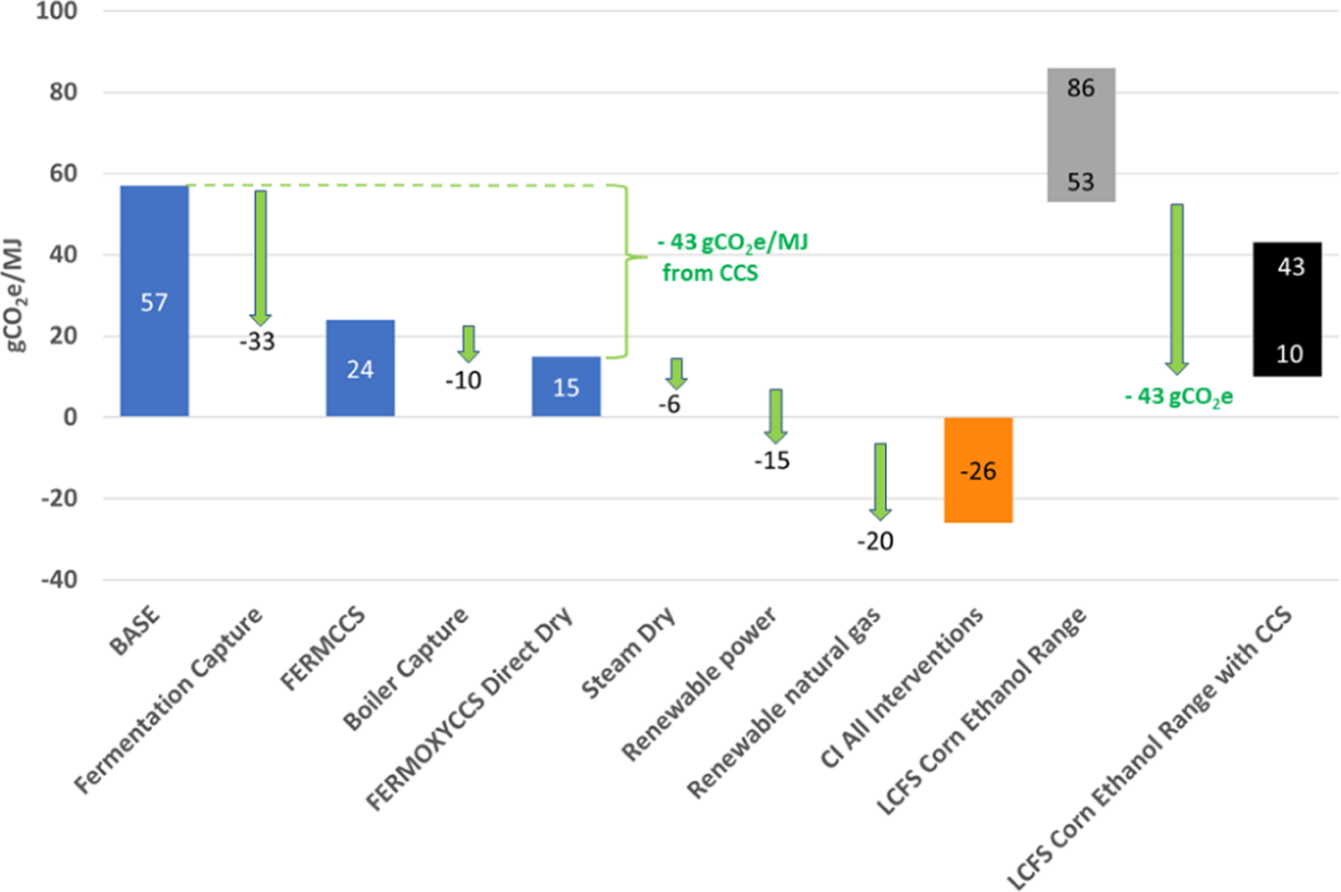
Carbon-negative ethanol can be achieved assuming all interventions. We adjust the range conservatively using the “net” CI reduction of the direct dry case which accounts for the additional power required for oxycombustion rather than the “gross” CO_2_ captured.

Finally, we assess the impact of all of these interventions combined on corn ethanol production. Figure 5 (left) illustrates a progression of emissions reductions from the BASE facility to include FERMCCS, FERMOXYCCS, steam drying, renewable electricity, and renewable natural gas. This system has a carbon intensity of −26 gCO_2_e/MJ. Without RNG, CI is −6 gCO_2_e/MJ, while without renewable electricity CI is −9 gCO_2_e/MJ. However, we note that some existing corn and sugar ethanol facilities already have a CI lower than the BASE scenario modeled here and, with the addition of CCS on fermentation and stack emissions, could achieve negative CI scores with fewer interventions. Figure 5 (right) illustrates this potential using the benchmark LCFS ranges discussed previously. Some of these facilities already utilize interventions such as renewable heat and power. For instance, the low-range CI score depicted by the gray bar (53 gCO_2_e/MJ) is utilizing landfill gas. Moreover, given lower CI electricity, the incremental improvement of an oxyfuel CCS system will be greater than the shift depicted below. Other CCS configurations (e.g., post-combustion capture) might achieve similar results. While carbon-negative sugarcane ethanol has been proposed,^14^ to our knowledge, this is the first time to demonstrate in the academic literature that corn ethanol production systems could result in net-negative emissions, removing CO_2_ from the atmosphere over the entire fuel life cycle.

#### 3.1.2 Cost of Emissions Abatement

**Figure 6:**
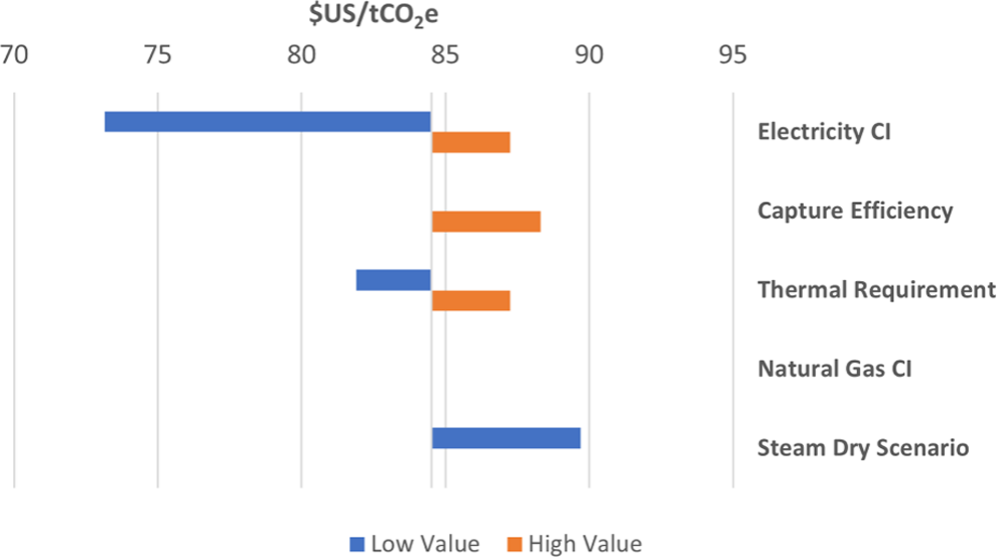
Sensitivity of carbon abatement costs to CI sensitivity scenarios. The alternative steam dry configuration is presented here as a sensitivity.

Any change in CI of the ethanol facility also results in a change in cost of carbon abatement for most cases, as both the BASE and FERMOXYCCS CI scores are affected. CAPEX and OPEX may be altered, as well as the distribution of costs over shifting relative CI reductions between BASE and FERMOXYCCS. The tested sensitivities primarily impact costs related to boiler capacity, ASU and CPU energy demand, and CO_2_ transport and storage. A summary of unit cost of emissions abatement sensitivities is shown in Figure 6.

The electricity CI sensitivity impacts the relative CI difference between BASE and FERMOXYCCS primarily by impacting carbon emissions associated with additional power requirements for the ASU and CPU. The low emissions case lowers the abatement cost to $73/ton CO_2_e, while the high emissions case increased the abatement cost to $87/ton CO_2_e. Notably, the low CI electricity case reduces the CO_2_ avoidance cost of the oxyfuel boiler component to $137/tCO_2_e. Electric grid decarbonization or purchase of renewable power (at a similar cost) can contribute to greater cost competitiveness of oxycombustion relative to post-combustion capture.

Low CO_2_ capture efficiency trades off lower CO_2_ clean-up and handling costs with lower overall abatement. Because costs in this case are spread over a smaller magnitude of CO_2_ reduction, the cost of emissions abatement increases to $88/t CO_2_e.

The change in thermal energy requirement has a dynamic effect on both costs and the emissions differential between the BASE and FERMOXYCCS scenarios. OPEX is positively correlated with the thermal requirement, in both BASE and FERMOXYCCS. In BASE, this is entirely fuel cost. In FERMOXYCCS, ASU and CPU capacity CAPEX and OPEX power demand are also affected, as well as CO_2_ handling costs. Boiler emissions increase or decrease in the BASE scenario in the high and low cases. Captured boiler emissions increase or decrease in the FERMOXYCCS scenario. Boiler capture leakage (2%) alters the relative abatement between the two cases. Upstream natural gas emissions are altered in both cases, but the impact is equivalent and does not affect the unit cost. In the low thermal energy requirement case, the cost of CO_2_ abatement decreases to $82/t CO_2_e while in the high thermal energy case, the cost increases to $87/t CO_2_e.

The upstream CI of natural is a fixed component and equivalent in both BASE and FERMOXYCCS cases in both the high and low sensitivity tests. As such, the unit cost of abatement is unaltered. Real-world costs for low-CI RNG are likely to be greater than conventional natural gas. While this would impact MESP, it would have no effect on the unit cost of abatement in the sensitivities as tested here because these costs would be equivalent in both BASE and FERMOXYCCS.

In the alternative steam dry scenario, the cost structure of CO_2_ abatement for FERMOXYCCS has significant differences to the direct dry BASE case. In this scenario, the boiler is sized larger to accommodate combustion of all natural gas for steam production. There are increased CAPEX costs for the larger boiler and increased demand on the ASU and CPU in FERMOXYCCS to handle both more fuel throughput in the boiler and greater volumes of CO_2_ in the capture stream. CO_2_ transport and storage cost OPEX increases, as well. Although this configuration results in a much lower overall CI, the cost of carbon abatement increases by approximately 6% relative to the direct dry FERMOXYCCS. The cost of carbon abatement is estimated to be $90/tCO_2_e. (More on the steam dry case can be found in SI S1.2 & S2.2).

#### 3.1.3 CAPEX and OPEX Sensitivities

Here we test the sensitivity of the MESP of the FERMOXYCCS system to variation in key CAPEX and OPEX assumptions. We tested CAPEX sensitivities only on the major components unique to FERMOXYCCS system relative to the BASE system. We apply a ±20% variation to the oxyfuel boiler, CPU, and ASU quoted costs before scaling factors for installation, equipment size, and cost index adjustments are applied. Similarly, feedstock, utilities, labor, and co-product revenues are the largest contributors to OPEX, with each category representing >10% of total operating costs. We apply a ±20% variation to base year costs to test the impact on the MESP relative to capital costs.

**Figure 7:**
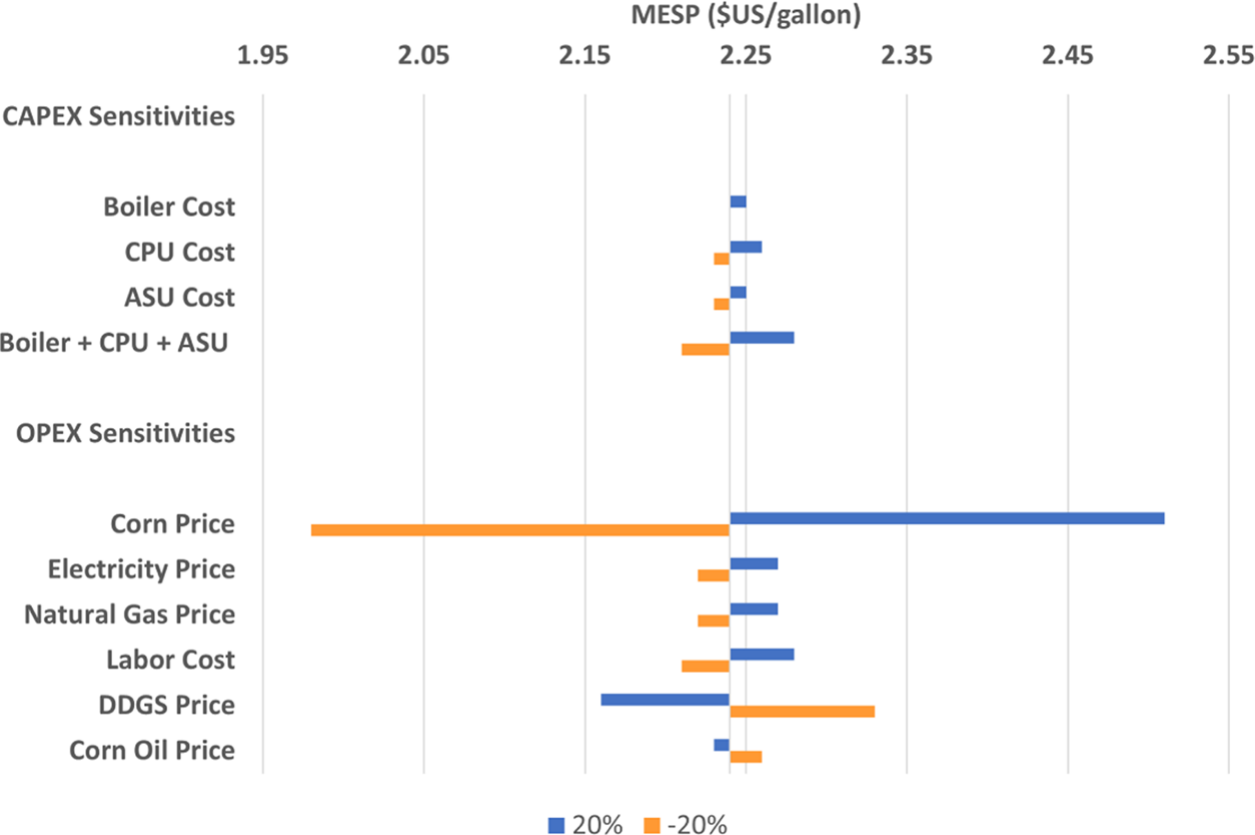
Sensitivity of MESP to a ±20% adjustment of CAPEX and OPEX assumptions.

The sensitivity of the MESP ($2.24/gallon) to capital costs is modest. Individual CAPEX components move the MESP by less than 1%. The combined sensitivity on the oxyfuel boiler, CPU, and ASU results in MESP ranging between $2.21 and $2.28/gallon. Electricity and natural gas both individually impact MESP by −0.9 to 1.3% yielding ranges between $2.22 and $2.27/gallon. Labor has a similar impact yielding MESP between $2.21 and $2.28/gallon. The most significant impacts result from feedstock price sensitivity and the selling price of the DDGS co-product, yielding MESP in the ranges of $1.98–$2.51/gallon (±12%) and $2.16–$2.33/gallon (±4%), respectively (Figure 7).

#### 3.1.4 Impact of Policy Support on MESP

Several state-level low-carbon fuel policies currently enacted in the U.S. have played a substantial role in the development of new low-carbon fuel projects. The California LCFS, in particular, has incentivized improvements in fuel CI in existing and proposed conventional ethanol facilities, as evidenced by the influx of program applicants and a steady trend in declining CI scores of approved production pathways.^77^ Thus, we elected to test the sensitivity of FERMOXYCCS MESP scenario to a low and high policy support market environment. We model policy incentives on the two most prominent policies in the U.S. context, California’s Low Carbon Fuel Standard (LCFS) and U.S. 45Q tax credit.

The LCFS is a performance-based standard that created a market for alternative fuel producers to sell avoided emissions credits. These credits are calculated based on the difference in CI between the alternative fuel and a state-mandated threshold for the average CI of fuels sold in the state. These credits can be sold to obligated fuel producers participating in the market such that fuels exceeding the CI threshold are brought into compliance. The gCO_2_e/MJ differential is converted to credits functionally equivalent to “tonnes of CO_2_e avoided” based on the energy content of volumes of fuel sold into the market. As of 2022, the CI threshold for gasoline (for which ethanol is a substitute) is 89.5 gCO_2_e/MJ. The modeled FERMOXYCCS facility would produce 244,530 credits per year based on a production of 38.9 MMgal/yr (∼3.2 billion MJ). See SI S4 for the LCFS credit calculation equations. Between July 2021 and May 2022, LCFS credit prices fell from $187 to $115 per tonne. Informed by this, we model a low policy support scenario at a credit price of $100/tonne and a high policy support scenario credit price of $200/tonne.

**Figure 8:**
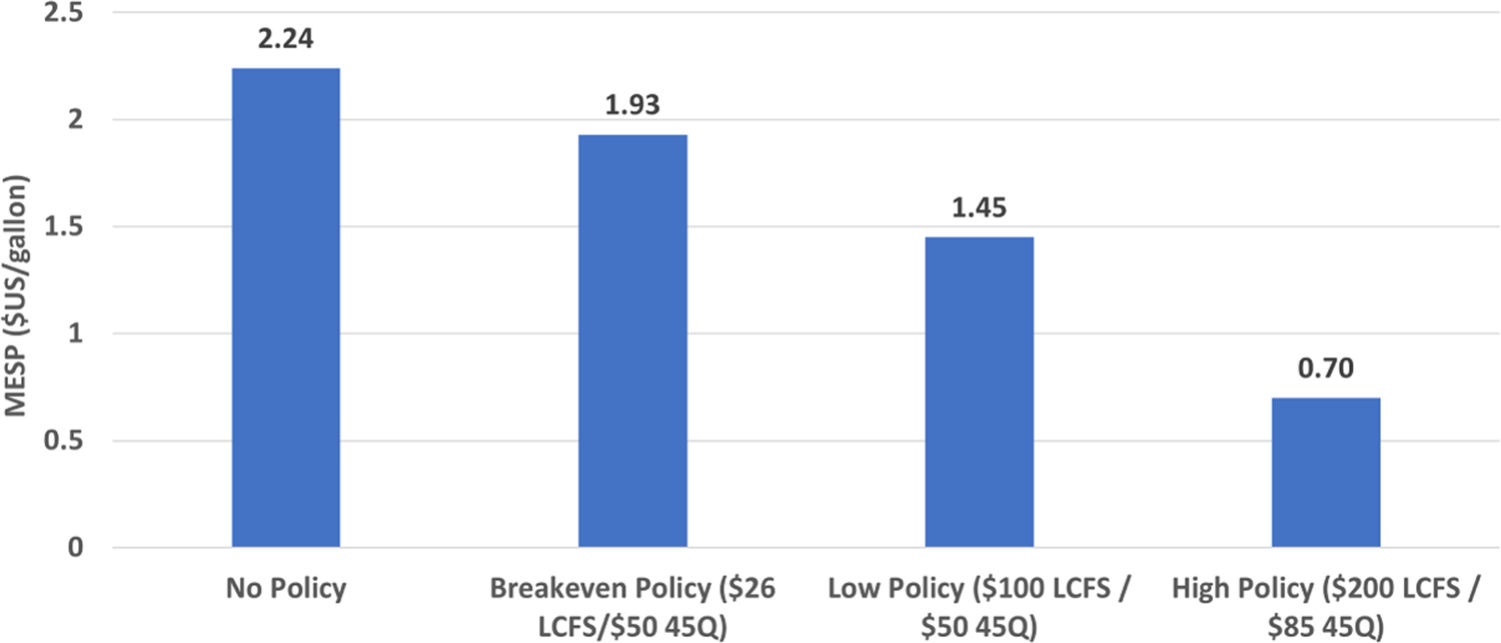
Sensitivity of MESP to policy support. LCFS = California Low-carbon Fuel Standard, 45Q = U.S. 45Q Tax Credit.

Fuel projects that incorporate CCS can also participate in the federal U.S. 45Q tax program. This policy stacks with LCFS revenues. U.S. 45Q is intended to incentivize carbon capture projects which result in permanent sequestration or utilization. As of May 2022, the highest incentive was for geologic sequestration, which awards a $50/ton credit for the first 12 years of operation. We model this value stacked with the LCFS in our low policy support scenario. In our high policy support scenario, we model an increase in the tax credit consistent with recent legislative adjustments to U.S. 45Q, increasing the credit to $85/ton. The modeled FERMOXYCCS facility would capture and sequester 139,432 tCO_2_e/year. The resulting MESP for the stacked low policy support case is $1.45/gal. While the high policy support case reduces the MESP to $0.70/gal. Holding the U.S. 45Q credit fixed at $50/tCO_2_, we also varied the LCFS credit to find the breakeven value with the BASE case (MESP = $1.93/gal). Breakeven occurs at an LCFS credit price of $26 per tonne (Figure 8).

#### 3.1.5 Discussion

Ethanol continues to play an important role as the most ubiquitous biofuel alternative to gasoline. The industry has the potential to play an even greater role in decarbonizing the transport sector through continued improvements in life cycle emissions. Decarbonization of light transport and performance-based low-carbon fuels policy incentives may soon favor electrification over liquid fuels. Nonetheless, low-carbon ethanol can serve as an important low-carbon platform in other market segments where policy support for CI performance exists such as sustainable aviation fuels or where it may soon exist, such as the chemicals and polymers industries.^78^ There is ample runway to further improve the CI of existing capacity and reduce the costs of doing so while maintaining the cost and CI competitiveness of ethanol as a sustainable transportation fuel. We are mindful of potential limits to the sustainable utilization of first-generation (food-based) crops for fuel production which will depend on the extent to which agricultural yields can meet increasing demand without deleterious effects on land and food systems. However, the findings herein are generally applicable to ethanol production from many potential feedstocks with lower sustainability risk and greater CI reduction potential than conventional corn. Applied to existing sugarcane and emerging cellulosic supplies of feedstock, the carbon removal potential of the ethanol industry is substantial.

The “low-hanging fruit” for corn ethanol refineries remains integration of CCS to capture and store biogenic CO_2_ from the fermentation process. This analysis along with other studies and commercial projects has demonstrated the technical and economic potential of this option. The low cost of CO_2_ capture from fermentation relative to other CO_2_ sources can help to facilitate learnings on carbon management and play a role in the development of a rapidly growing carbon removal and storage industry. Even so, conventional ethanol with fermentation CCS is still far from carbon neutral. If ethanol is to continue to play a role in deep decarbonization and achieving climate stability targets, the CI of ethanol must continue to be driven down.

Process and fuel interventions that address fossil emissions associated with heat and power represent another promising opportunity to realize very low-carbon or even carbon-negative ethanol. Several options to address those emissions have been analyzed here. CCS on oxyfuel boiler and fermentation emissions can reduce ethanol carbon intensity by as much as 71% at prices under $100/ton CO_2_e. Moreover, sensitivity analysis has demonstrated that in combination with other interventions such as renewable energy and fuel switching to bio-derived fuels, conventional ethanol refineries can produce carbon-neutral or even negative fuel, potentially at profit under existing policy support.

Integration of oxyfuel combustion and CCS at ethanol facilities will present unique challenges and opportunities for learnings. Further research, process engineering design, and demonstration will be necessary to understand the full potential and compare with the technical and economic feasibility of alternative interventions. Further research could investigate alternatives to oxyfuel combustion such as increased electrification of refinery heat demand, improved efficiency, pre-combustion and post-combustion CCS configurations, and alternative bio-heat production (e.g., anaerobic digestion) such that additional synergies and opportunities may be realized. Each could present new opportunities to further reduce the CI of conventional biofuels.

## Supporting Information Available

Mass and energy balance of ethanol plant (S1); block-flow representation of ASPEN model (Figure S1); corn composition (Table S1); air separation unit modeling parameters (Table S2); carbon balance for steam and direct dry cases (Table S3); Aspen results summary (Table S4); LCA assumptions and extended LCA analysis (S2); life cycle inventory (Table S5); extended LCA results (Figure S2); CO_2_ capture cost model (S3); review of costs for the air separation unit (Table S6); cost versus capacity power regression analysis (Figure S3); and California Low Carbon Fuel Standard credit calculations (S4) (PDF)
