# Supplementary material for: Cost and Life Cycle Emissions of Ethanol Produced with an Oxyfuel Boiler and Carbon Capture and Storage
Source: Environ Sci Technol. 2023 Mar 21;57(13):5391–403. doi: 10.1021/acs.est.2c04784 (PMC10077580; doi:10.1021/acs.est.2c04784)
Supplement: Supplementary file 1 — es2c04784_si_001.pdf [file es2c04784_si_001.pdf]

Supplemental Information (SI) for Research Article

Oxyfuel combustion with carbon capture and sequestration to produce low-carbon ethanol

John Dees[a], Kafayat Oke[b], Hannah Goldstein[c], Sean T. McCoy[b], Daniel Sanchez[d], A.J. Simon[c], Wenqin Li[c]*

[a]Energy and Resources Group, University of California, Berkeley, 345 Giannini Hall, Berkeley, CA, 94720, USA

[b]Department of Chemical and Petroleum Engineering, University of Calgary, 750 Campus Dr NW, Calgary, AB T2N 4H9, Canada

[c]Lawrence Livermore National Laboratory, 7000 East Avenue, Livermore, CA, 94550, USA

[d]Environmental Science, Policy, and Management (ESPM), University of California, Berkeley, 130 Mulford Hall #3114, Berkeley, CA, 94720, USA

*Correspondence and requests for materials should be addressed to Dan Sanchez:

Department of Environmental Science, Policy, & Management, UC Berkeley, 130 Mulford Hall, #3114, Berkeley, CA 94720, USA Email: sanchezd@berkeley.edu

Contains four text sections in 8 pages, 6 tables, and 3 figures.

S1 Mass and Energy Balance

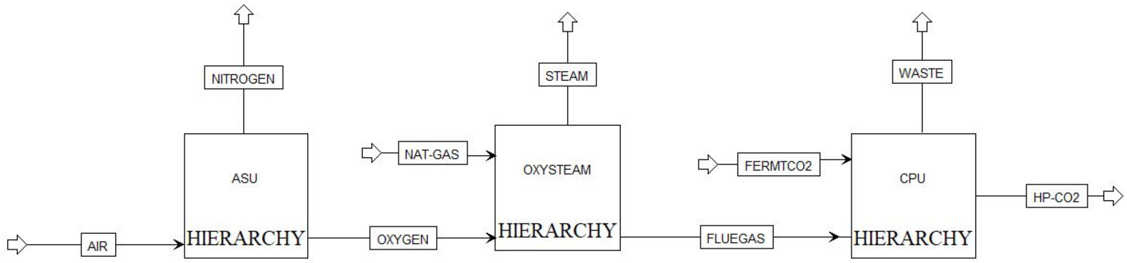

Figure S1: Block flow representation/ Scope of ASPEN Model

S1.1 Material balance

The material balance of the quantity of CO2 capturable from a 40 million gallon per year denatured (2.5 vol%) ethanol plant, running 358 days/annum is stated below. The composition of corn used is reviewed from literature sources [1]–[4] and given in Table S1. Fermentation is assumed to have 93.2% conversion efficiency, while liquefaction and saccharification conversion efficiency and ethanol recovery is 99%. Corn is assumed to compose of 40.52% carbon. Density of ethanol is 0.79 kg/L. The reaction equations are stated below.

Liquefaction of starch to maltose

$$2(C_6H_{10}O_5)_n + nH_2O \xrightarrow{\alpha - amylase} nC_{12}H_{22}O_{11}$$

Saccharification of maltose to glucose

$$C_{12}H_{22}O_{11} + H_2O \xrightarrow{glucoamylase} 2C_6H_{12}O_6$$

Fermentation of glucose to ethanol

$$C_6H_{12}O_6 \xrightarrow{yeast} 2C_2H_6O + 2CO_2$$

From the above equations, 1kg of starch produces 1.06 kg of maltose and 1kg of maltose produce 1.05 kg of glucose. 1kg of glucose produces 0.51 kg of ethanol and 0.49 kg of CO_2.

S1.2 Energy balance

Mueller (2008) reported the thermal energy requirements of a dry grind ethanol mill in US as 8.08 MJ/L [7]. In this work, we have modelled the steam requirement of a 40 million gallon per year (189 million litre per year) ethanol plant supplied by oxyfuel combustion of natural gas, capturing the CO_2 produced during the combustion and fermentation step using ASPEN plus V11. Peng-Robinson (PENG-ROB) equations of state is the property methods selected.

Table S1: Corn composition

Component	%wt basis
Starch	62.9
Glucose	1.70
Cellulose/Hemicellulose	7.2
Protein	8.1
Oil	3.8
Ash	1.3
Water	15.0
**Total**	**100.0**
Shelled corn (lb/bu)	56

Table S2: ASU modelling parameters

	**Scenario 1 – Direct Dry**	**Scenario 2 – Steam Dry**	
**Parameter**	**Value**	**Value**	**Unit**
Flowrate	25100	40635	Nm^3/hr
Pressure	1	1	bar
Temperature	15	15	^oC
N_2	78.1	78.1	%
O_2	21	21	%
Ar	0.9	0.9	%
Compressor efficiency	85	85	%
O_2 purity	95	95	%
O_2 pressure	1.2	1.2	bar

Table S3: Carbon balance for both cases

**Scenario 1 – Direct Dry**	**Carbon in**	**Carbon out**

Source		Flowrate	%C	C in kg/hr		Flowrate	%C	C in kg/hr
Fermentation	Corn	40951	40.52%	16595	EtOH	13701	52%	7143
					DDGS	11514	49%	5642
					Corn oil	313	76%	238
Oxyfuel	Natural gas			1383	Oxy vents	5.5	27%	1.5
					Ferment vents	6.5	27%	1.8
					CO_2 product	18145	27%	4952
**Total**				**17978**				**17978**
**Scenario 2 – Steam Dry**	**Carbon in**				**Carbon out**			
Source		Flowrate	%C	C in kg/hr		Flowrate	%C	C in kg/hr
Fermentation	Corn	40951	40.52%	16595	EtOH	13701	52%	7143
					DDGS	11514	49%	5642
					Corn oil	313	76%	238
Oxyfuel	Natural gas			2239	Oxy vents	9.2	27%	2.52
					Ferment vents	6.5	27%	1.78
					CO_2 product	21278	27%	5807
**Total**				**18834**				**18834**

Table S4: Results summary

**Parameter**	**Scenario 1 – Direct Dry**	**Scenario 2 – Steam Dry**
Air flow rate (t/d)	776	1256
O_2 flow rate (t/d)	189	306
N_2 flow rate (t/d)	873	950
Natural gas flow rate (t/d)	46	74
Steam flow rate (t/d)	779	1265
CO_2 product flow rate (t/d)	435	511

ASU SER (kWh/t O_2)	196	196
CPU SER (kWh/t CO_2)	115	117
Combustion temperature (^oC)	1053	1053

S2 LCA Assumptions and Extended Analysis

S2.1 Life Cycle Inventory

Table S5: LCA Inventory. Aggregate emissions factors will differ somewhat from GREET.net assumptions. Raw data was extracted and tabulated independently in an Excel model. We use AR5 GWP factors for CH_4 and N_2O whereas GREET uses AR4 factors.

Input	Qty	gCO_2e	Source
Corn (g)	1.12E2	3.70E1	GREET.net 2019
Alpha amylase (g)	3.17E-2	3.84E-2	GREET.net 2019
Gluco amylase (g)	6.82E-2	3.77E-1	GREET.net 2019
Yeast (g)	3.46E-2	8.69E-2	GREET.net 2019
Process water (gal)	3.42E-2	0.00	GREET.net 2019
Sulfuric acid (g)	5.86E-2	2.59E-3	GREET.net 2019
Ammonia (g)	2.25E-1	5.62E-1	GREET.net 2019
Sodium Hydroxide (g)	2.82E-1	5.65E-1	GREET.net 2019
Calcium Oxide (g)	1.34E-1	1.72E-1	GREET.net 2019
Natural gas (boiler) – Direct dry (Btu)	2.22E2	1.48E1	GREET.net 2019 NA Shale/Conventional + Utility boiler
Natural gas (dryer) – Direct dry (Btu)	1.38E2	9.10E0	GREET.net 2019 Shale/Conventional + Dryer
Natural gas (boiler) – Steam dry (Btu)	3.60E2	2.41E1	GREET.net 2019 Shale/Conventional + Utility boiler
Natural gas (oxyfuel boiler) – Direct dry (Btu)	2.22E2	1.48E1	GREET.net 2019 + Aspen
Natural gas (oxyfuel boiler) – Steam dry (Btu)	3.60E2	2.41E1	GREET.net 2019 + Aspen
Electricity – BASE (Btu)	3.08E1	6.02E0	GREET.net 2019 MROW Distributed
Additional Electricity FERMCCS (Btu)	1.41E1	2.76E0	GREET.net 2019 MROW Distributed
Additional Electricity FERMOXYCCS (Btu)	3.39E1	6.63E0	GREET.net 2019 MROW Distributed
Additional Electricity SD-FERMOXYCCS (Btu)	4.66E1	9.11E0	GREET.net 2019 MROW Distributed
**Output**			
Ethanol (MJ)	1.00		Aspen Yield
DDGS (g)	3.15E1	-1.16E1	Aspen Yield + GREET Displacement EF
Corn oil (g)	8.57E1	-2.10E-1	Aspen Yield + GREET Displacement EF

S2.2 Extended LCA Results

We chose to focus our analysis on a conservative subset of cases wherein a direct drying system is used to dry the DDGS co-product. In the direct dry configuration, only 62% of the natural gas is combusted in the boiler, thus only the CO_2 emissions from the boiler fraction of fuel combustion is available to the capture system. However, we model an alternative scenario where all natural gas is combusted in the boiler and DDGS is dried indirectly via the steam dry configuration. In **Figure S2,** the direct dry cases BASE, FERMCCS, and FERMOXYCSS reported in the manuscript are on the far left. BASE-RNG, FERMCCS-RNG, and FERMOXYCCS-RNG model the same direct dry cases but substitute renewable natural gas (RNG) from upgraded landfill gas (described in manuscript sensitivity analysis) for conventional natural gas. The six cases on the right-hand side of the figure with the SD- designation represent the life cycle GHG intensity of the “steam dryer” configuration of each of the direct dry scenarios. The net CI reduction between FERMOXYCCS and SD-FERMOXYCCS is -6 gCO_2e/MJ. The steam dry configuration enables an additional 8 gCO_2e/MJ of avoided emissions relative to the direct dry case. However, the process also generates a little over 2 gCO_2e/MJ more emissions from the electric grid, as additional power is required to support the ASU and CPU due to more fuel in oxyfuel boiler.

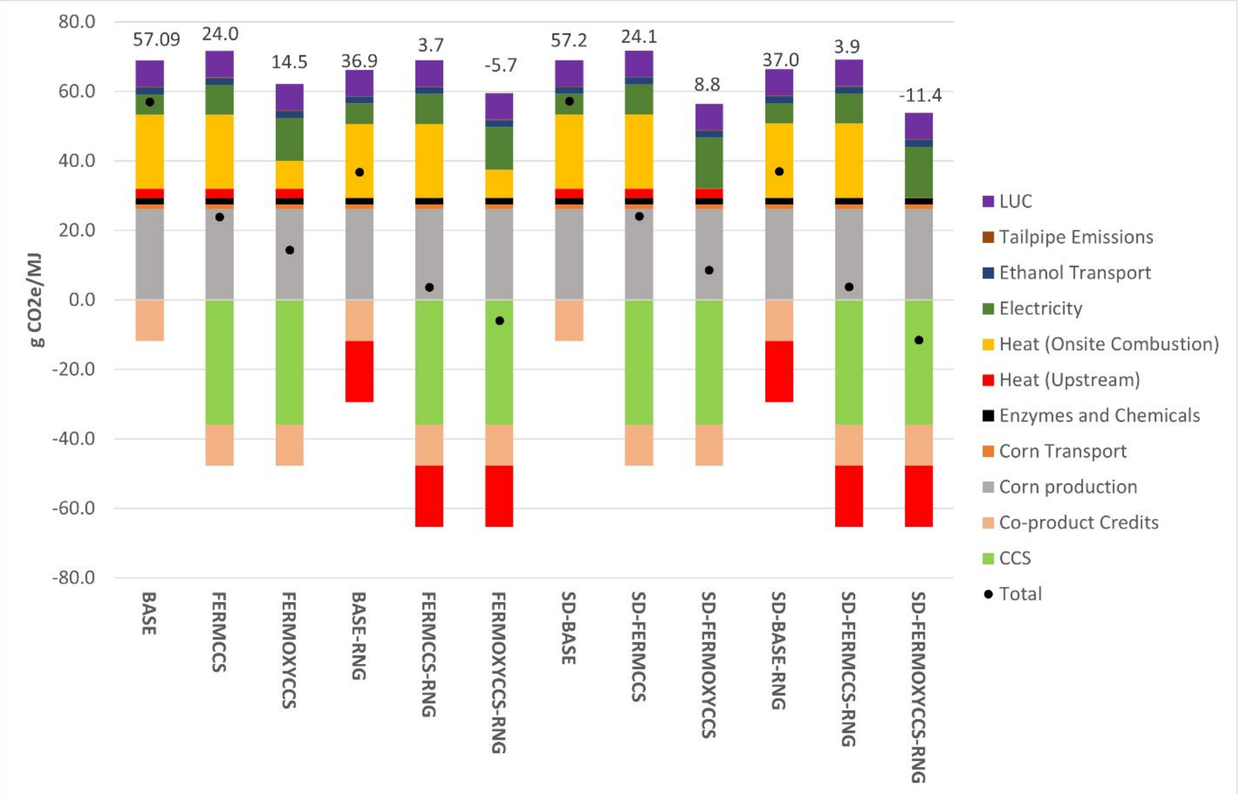

Figure S2. Life cycle carbon intensity (CI) of twelve ethanol process configurations BASE = Baseline facility with direct drying of DDGS, FERMCCS = CCS on fermentation gas only, FERMOXYCCS = Oxyfuel boiler added with CCS on both fermentation and boiler flue gas streams, CCS = Carbon Capture and Sequestration, RNG= Renewable natural gas substituted for conventional NG, SD – Steam Dry configuration, i.e., all natural gas fuel is combusted in boiler, LUC = Land Use Change.

S3. CO_2 Capture Cost Model

S.3.1. Air separation unit (ASU)

The cost-to-capacity method for computing order-of-magnitude cost estimates meaning using known cost and capacity of an existing plant or equipment to calculate cost of a new plant or equipment. The calculated cost from this method gives a Class 4 or 5 estimate as specified by Association for the Advancement of Cost Engineering (AACE) International [5].

The cost to capacity's concept is that cost of different sizes of equipment or facilities using similar technology vary nonlinearly which is associated with economies of scale. That is, it tends to cost less to build larger plants per unit capacity. The governing equation is given below [6].

$$C_2 = C_1 \left[\frac{S_2}{S_1}\right]^n \quad \text{...1}$$

Where, C_1 = known capital cost of the plant 1

C_2 = Required capital cost of the plant 2

S_1 = Capacity of the plant 1

S_2 = Capacity of the plant 2

n = Scaling exponent

The technology for oxygen separation from air is the cryogenic distillation. The equipment cost is the direct cost of the process equipment excluding labour, material, installation, direct, and indirect costs. Two sources in literature with corresponding base year are listed in table S6. The reference cost has been updated to 2020 dollars using the Chemical Engineering Plant Cost Index (CEPCI).

The relationship is given as [6]:

$$C_2 = C_1 \left[\frac{Cost\ Index\ in\ year\ 2}{Cost\ Index\ in\ year\ 1}\right] \quad \text{... 2}$$

Table S6: Reviewed ASU equipment cost (2020) and capacities

Label	Source	Cost year	Capacity (tonnesO_2/day)	Updated Equipment Cost (M$2020)
A	NETL-2019 [7]	2018	3665	$52.68
B	NETL-2019 [7]	2018	3687	$52.90
C	NETL-2019 [6]	2018	3954	$55.54
D	NETL-2019 [7]	2018	4186	$57.81
E	NETL-2019 [7]	2018	4288	$58.80
F	NETL-2012 [8]	2007	11681	$126.35
G	NETL-2012 [8]	2007	12798	$133.46
H	NETL-2012 [8]	2007	12955	$134.45

To determine the scaling exponent of the ASU, a power law scaling curve of cost vs capacity is plotted. Figure S3 shows the cost versus capacity power regression analysis. The exponent is calculated as 0.75. The suggested scaling exponent for ASUs given by "Quality Guideline for Energy System Studies – Capital Cost Scaling Methodology" QGESS [9] and Hamelinck *et al.* [10] are 0.70 and 0.75 respectively.

Therefore, the governing scaling equation for calculating the cost of cryogenic ASU using the capacity of oxygen produced as scaling parameter is:

$$ASU\ Equipment\ Cost\ (M_{2020}\ USD) = 0.1126\left[Capacity\left(\frac{tonne\ O_2}{day}\right)\right]^{0.75} \quad ...3$$

Therefore, for a cryogenic ASU capacity of 377 tpd O_2, the equipment cost is calculated as \$9.63M. According to Air Liquide Engineering and Construction technology 2021 handbook [11], the cost of a Sigma – Standard Air Separation Unit, which produces 110 to 380 tpd O2 up to 99.8% purity is between 5.37 – 9.67M\$2020. Based on this validation, ASU is scaled from Air Liquide cost with a scaling exponent of 0.75.

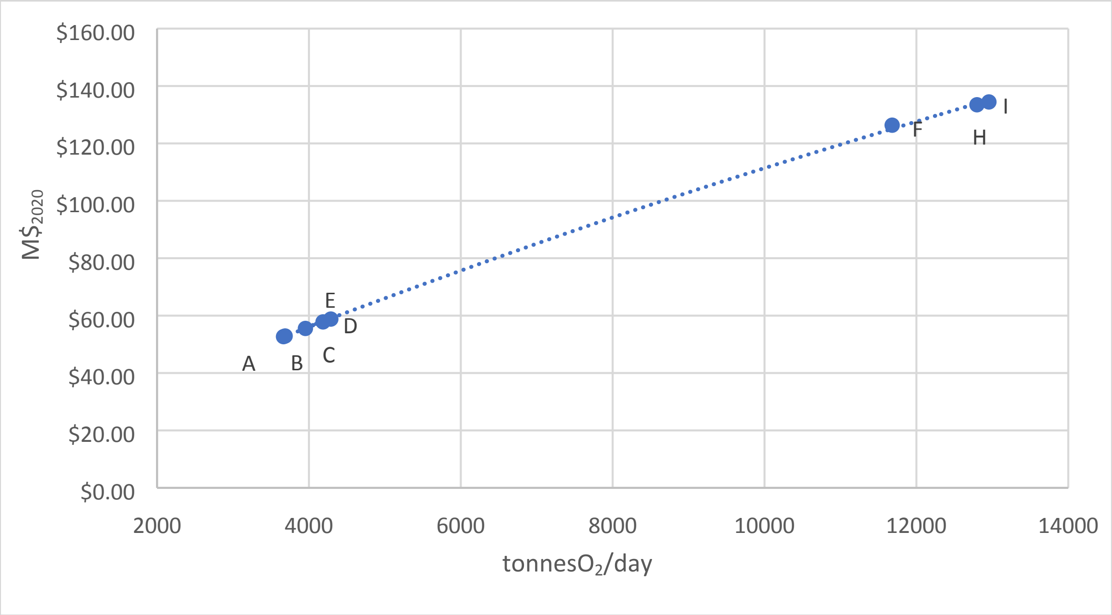

Figure S3: *Cost versus capacity power regression analysis*

S3.2. CO_2 purification unit (CPU)

The CPU was scaled directly from the Illinois Basin Decatur Project (IBDP) [11] with a scaling exponent of 0.8 for compression and dehydration equipment, and 0.6 for electrical transmission line, instrumentation, and controls.

S3.3 Oxyfuel boiler

There is very limited literature cost of oxyfuel utility boilers. For this analysis, the cost of oxyfuel boiler is based off the air combustion utility boiler. An installation factor of 4 is suggested for the additional modification. Such modifications include [12]:

- Oxyburners as opposed to normal burners
- Flue gas recycling ducts and fans
- Air preheater replaced by economizer
- Superheater and attemperators

S4. California Low-Carbon Fuel Standard (LCFS) Credit Calculations

The following formula is used to calculate credits and deficits generated under California's LCFS program. Deficit generators (sellers of fossil fuels whose fuels do not meet the CI standard) must purchase credits from credit generators (alternative fuel producers whose fuel has a CI lower than the CI standard) such that all deficits are cancelled by credits and the average CI of fuel sold in California meets the CI criteria for the current year. The formula below is adapted from **Title 17, California Code of Regulations (CCR), section §95486.1. "Generating and Calculating Credits and Deficits Using Fuel Pathways."**

$$\boldsymbol{Credits_i^{XD}\ or\ Deficits_i^{XD}(MT) = \left(CI_{standard}^{XD} - CI_{reported}^{XD}\right) \times E_{displaced}^{XD} \times C}$$

Where:

$\boldsymbol{Credits_i^{XD}\ or\ Deficits_i^{XD}(MT)}$ is either the number of LCFS credits generated (a zero or positive value), or deficits incurred (a negative value), in metric tons, by a fuel or blendstock under the average carbon intensity requirement for gasoline (XD = "Gasoline"), diesel (XD = "Diesel"), or jet fuel (XD = "jet fuel).

$\boldsymbol{CI_{standard}^{XD}}$ is the average carbon intensity requirement of the either gasoline, diesel, or jet fuel for a given year.

$\boldsymbol{CI_{reported}^{XD}}$ is the adjusted carbon intensity value of a fuel or blendstock in gCO2e/MJ

$\boldsymbol{E_{displaced}^{XD}}$ is the total quantity of gasoline, diesel, or jet fuel displaced in MJ

C is a factor used to convert credits to units of metric tons from gCO2e and has the value of:

$$C = 1.0 \times\ 10^{-6} \frac{(MT)}{(gCO2e)}$$

References

[1] A. McAloon, F. Taylor, and W. Yee, "Determining the Cost of Producing Ethanol from Corn Starch and Lignocellulosic Feedstocks," p. 44, 2000.

[2] R. Karuppiah, A. Peschel, I. E. Grossmann, M. Martín, W. Martinson, and L. Zullo, "Energy optimization for the design of corn-based ethanol plants," *AIChE J.*, vol. 54, no. 6, pp. 1499–1525, Jun. 2008, doi: 10.1002/aic.11480.

[3] J. R. Kwiatkowski, A. J. McAloon, F. Taylor, and D. B. Johnston, "Modeling the process and costs of fuel ethanol production by the corn dry-grind process," *Industrial Crops and Products*, vol. 23, no. 3, pp. 288–296, May 2006, doi: 10.1016/j.indcrop.2005.08.004.

[4] BBI International, "Corn Ethanol Industry Process Data: September 27, 2007 - January 27, 2008," NREL/SR-6A1-45152, 948744, Feb. 2009. doi: 10.2172/948744.
[5] "18R-97: Cost Estimate Classification System – As Applied in Engineering, Procurement, and Construction for the Process Industries," p. 7, 2020.
[6] G. P. Towler and R. K. Sinnott, *Chemical engineering design: principles, practice, and economics of plant and process design*, 2nd ed. Boston, MA: Butterworth-Heinemann, 2013.
[7] R. E. James III PhD, D. Kearins, M. Turner, M. Woods, N. Kuehn, and A. Zoelle, "Cost and Performance Baseline for Fossil Energy Plants Volume 1: Bituminous Coal and Natural Gas to Electricity," NETL-PUB-22638, 1569246, Sep. 2019. doi: 10.2172/1569246.
[8] Marc J. Turner, Mark Woods, Scott Chen, Robert D. Brasington, John L. Haslbeck, Charlie Zhang, "Advancing Oxycombustion Technology for Bituminous Coal Power Plants: An R&D Guide," National Energy Technology Laboratory (NETL)., DOE/NETL-2010/1405, Apr. 2012.
[9] Marc J. Turner, Lora L. Pinkerton, "Quality Guideline for Energy System Studies – Capital Cost Scaling Methodology" QGESS," National Energy Technology Laboratory (NETL)., DOE/NETL-341/013113, Jan. 2013.
[10] C. Hamelinck, A. Faaij, H. Denuil, and H. Boerrigter, "Production of FT transportation fuels from biomass; technical options, process analysis and optimisation, and development potential," *Energy*, vol. 29, no. 11, pp. 1743–1771, Sep. 2004, doi: 10.1016/j.energy.2004.01.002.
[11] Air Liquide Engineering and Construction, "Technology Handbook," Jun. 2021. Accessed: Oct. 21, 2021. [Online]. Available: https://www.engineering-airliquide.com/technology-handbook
[12] T. Uchida, T. Goto, T. Yamada, T. Kiga, and C. Spero, "Oxyfuel Combustion as CO2 Capture Technology Advancing for Practical use - callide Oxyfuel Project -," *Energy Procedia*, vol. 37, pp. 1471–1479, 2013, doi: 10.1016/j.egypro.2013.06.022.
